# Neurotrophic Keratopathy in Systemic Diseases: A Case Series on Patients Treated With rh-NGF

**DOI:** 10.3389/fmed.2022.920688

**Published:** 2022-05-30

**Authors:** Alessandro Meduri, Giovanni William Oliverio, Antonio Valastro, Claudia Azzaro, Umberto Camellin, Francesco Franchina, Leandro Inferrera, Anna Roszkowska, Pasquale Aragona

**Affiliations:** ^1^Department of Biomedical Sciences, Ophthalmology Clinic, University of Messina, Messina, Italy; ^2^Department of Medical, Surgical Sciences and Health, Eye Clinic, University of Trieste, Trieste, Italy; ^3^Department of Ophthalmology, Faculty of Medicine and Health Sciences, Andrzej Frycz Modrzewski Krakow University, Kraków, Poland

**Keywords:** neurotrophic keratopathy, rh-NGF, neurotrophic keratitis, Cenegermin, nerve growing factor

## Abstract

**Purpose:**

To evaluate the prevalence, clinical ocular presentation and corneal healing in moderate and severe neurotrophic keratopathy (NK) caused by systemic diseases and treated with rh-NGF.

**Setting:**

Department of Biomedical and Dental Sciences and Morphofunctional Imaging, Ophthalmology Clinic, University of Messina, Italy.

**Design:**

Retrospective observational study of case series.

**Materials and Methods:**

In this retrospective observational study 11 patients (five female and six males) aged from 24 to 88 years (55.4 ± 21.3 years) with moderate and severe NK caused by systemic diseases were enrolled. The VAS questionnaire was dispensed. The ocular examination comprised slit lamp evaluation, ocular surface assessment with Keratograph 5M (Oculus, Germany), corneal sensitivity with Cochet-Bonnet esthesiometer (Lunneaux, France) and corneal thickness measurement with AC-OCT (DRI, Triton, Topcon, Japan). The underlying systemic causes of NK were determined.

**Results:**

The main cause of NK was post-neuroma surgery (36%), followed by diabetes (18%). The remaining causes were rheumatoid arthritis (9%), post-traumatic (9%), post-surgery (9%), atopia (9%), Graves' disease (9%). Seven eyes presented severe grade of NK with corneal ulcer and in four a moderate grade was registered. The rh-NGF (Cenegermin) was administered with a standard protocol one drop six times daily for 8 weeks. The complete healing of all corneal defects was registered at the end of the treatment.

**Conclusions:**

The post-neuroma surgery was the most common cause of NK and severe grade was clinically more represented. The rh-NGF proved effective to promote corneal recovery with all defects healed after the treatment.

## Introduction

Neurotrophic keratopathy (NK) is a degenerative corneal disease that affects the health and integrity of the ocular surface, resulting from impairment of corneal nerves that causes alterations in their sensory and trophic function (1). When the corneal epithelium is damaged, a coordinated and collaborative system of communication between epithelial and neuronal cells is required to promote the resynthesis of the damaged matrix, cell migration, and the restoration of architectural integrity (2, 3). As a result of permanent impairment of epithelial repair, the exposed stroma becomes subject to enzymatic deterioration, melting, and in severe forms perforation, which are all characteristics of NK (2). NK is defined as a rare disease with a prevalence estimated between 1.6 and 4.2/10,000 individuals. However, the recent studies demonstrated that this condition is commonly underestimated (4).

The etiology of corneal nerves alteration in NK could be linked to numerous ocular or systemic conditions (4, 5).

The main local causes reported are herpetic infections, chemical injuries, while the corneal surgery could directly damage the corneal nerves (5).

Although the etiology of neurotrophic keratitis is commonly related to primary ocular diseases, there are several systemic or genetic diseases, and central nervous disorders that may underlie this corneal affection (4–6).

Recent knowledge in pathogenesis of NK and the introduction of topical recombinant human Nerve Growth factor (rh-NGF) has significantly changed the natural history of the disease (1, 2).

The purpose of this study is to analyze the prevalence of moderate and severe NK resulting from systemic diseases in affected patients treated in our center with rh-NGF, aiming to identify the most frequent cause and the grade of corneal involvement. The additional aim is to assess the corneal healing process during the treatment.

## Materials and Methods

In this retrospective observational cohort study, 21 eyes of 21 patients with moderate and severe NK treated with rh-NGF between January 2017 and March 2020 at Excellence Regional Center for Ocular Surface diseases of the Ophthalmology Clinic of the University Hospital of Messina were enrolled. The study was conducted with respect of tenets of Declaration of Helsinki and obtained approval of the Ethical Committee of the University Hospital of Messina. For the study purposes only patients with underlying systemic diseases that caused NK were included with the aim to determine the percentage of presentation of moderate and severe forms accordingly to the underlying pathology. Therefore, 10 patients were excluded from the study as they were affected by NK which had an ocular pathology as the primary cause. The enrolled sample comprised 11 patients with systemic diseases. Five patients were female and 6 were male and their age ranged from 24 to 88 years (mean 55.4 ± 21.3 years).

All patients underwent ocular examination that included slit lamp evaluation, ocular surface assessment with Keratograph 5M (Oculus, Germany), corneal sensitivity with Cochet-Bonnet esthesiometer (Lunneaux, France) and corneal thickness measurement with AS-OCT (DRI, Triton, Topcon). Dua classification for NK severity determination was used (1) and moderate form defined as persistent epithelial defects (PED) was differentiated from severe one (ulcer) in relation to the corneal involvement. The corneal surface was analyzed with Keratograph 5M using fluorescein staining.

Corneal sensitivity was measured in the center of the cornea three times with the Cochet-Bonnet esthesiometer and reported in filament length (cm). The average of the three measurements was used. In cases of severe NK, the thinnest point in the ulcer bed was measured using Swept source AS-OCT (DRI Triton, Topcon, Japan). The ocular discomfort was assessed using the Visual Analog Scale (VAS). All patients received Cenegermin drops (20 μg/ml) (Oxervate ^®^, Dompè, farmaceutici Spa, Milan, Italy) accordingly to the standardized protocol with one drop for six times daily for 8 weeks.

Corneal healing was defined as <0.5 mm fluorescein staining in the lesion area, according to REPARO 2 (7).

## Results

In a total of 21 patients, 11 (52.4%) presented NK related to systemic diseases, and 10 (47.6%) to ocular affections.

Furthermore, the group with underlying systemic causes was evaluated. The main cause of NK was post-neuroma surgery (36%), followed by diabetes (18%), previous surgery (9%), complications of Graves' disease (9%), previous trauma (9%), ocular complications of rheumatoid arthritis (9%) and severe atopic dermatitis (9%).

**Figure 1:**
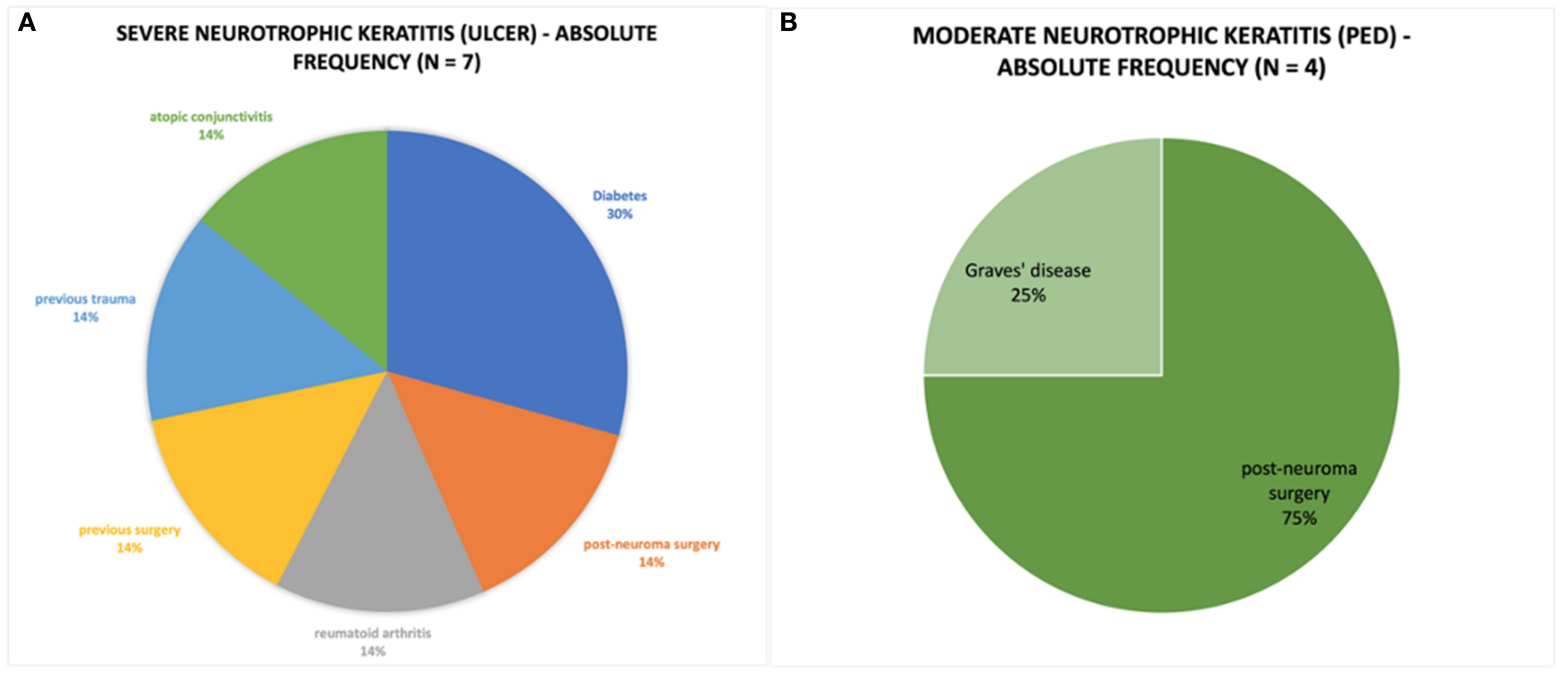
**(A)** Pie chart represent the absolute frequency of cases of severe neurotrophic keratitis caused by systemic pathology. **(B)** Pie chart representing the absolute frequency of cases of moderate neurotrophic keratitis caused by systemic pathology.

Severe NK (ulcer) was observed in seven patients (mean age 62.3 ± 21.7 years) and moderate NK (PED) in four patients (mean age 43.3 ± 16.1 years). The causes and severity distribution of NK are shown in Figure 1. Moderate NK was related to post-neuroma surgery (75%) and Graves' disease (25%), whereas severe NK to diabetes (30%), and other causes (Figure 1). Total VAS score was 20.27 ± 4.11 mm before the treatment, 20.98 ± 3.63 mm after 4 weeks and 10.92 ± 7.19 mm after 8 weeks (Table 1). Corneal sensitivity improved in all eyes. The changes of sensitivity and VAS score from baseline to 8 weeks after rh-NGF administration are shown in (Tables 1, 2).

**Table 1:** Corneal sensibility in severe and moderate neurotrophic keratitis.

**Presentation**	**Baseline**	**4 weeks**	**8 weeks**
Severe NK	1.9 ± 1.8	4.5 ± 0.7	4.6 ± 0.5
Moderate NK	1.1 ± 0.5	3.3 ± 2	3.9 ± 1.9
Total	1.6 ± 1.5	3.6 ± 1.7	4.25 ± 1.3

*All data are reported as mean ± standard deviation*.

**Table 2:** Visual analog scores in severe and moderate neurotrophic keratitis.

**Presentation**	**Baseline**	**4 weeks**	**8 weeks**
Severe NK	23.36 ± 4.38	21.28 ± 3.87	11.91 ± 7.80
Moderate NK	20.27 ± 4.11	20.98 ± 3.63	10.92 ± 7.19
Total	21.81 ± 4.24	21.13 ± 3.75	11.41 ± 7.49

*All data are reported as mean ± standard deviation*.

As to the corneal surface recovery after 4 weeks of the treatment a complete healing was registered in 100% of ulcers and 50% of PED. After 8 weeks a complete corneal recovery was observed in all patients.

Corneal thinnest location pachymetry in patients with severe NK was 285.25 ± 71.83 μm before treatment, 385.5 ± 16.33 μm at 4 weeks, and 448.25 ± 37.71 μm at 8 weeks (Table 2).

## Discussion

In our series there was a higher prevalence of NK resulted from several systemic diseases (52.4%); whereas the ocular causes of NK were related mainly to herpetic infections (47.6%).

Systemic diseases such as diabetes, multiple sclerosis, central nervous system diseases, genetic syndromes and autoimmune diseases could be associated with NK (1, 4–12).

A recent epidemiologic study on 335 patients showed that central nervous systems diseases followed by diabetes are the main causes of NK due to the systemic conditions (13).

In our study, the main systemic causes of NK were post neuroma surgery (36%) and diabetes (18%) and such finding confirms these recently published data.

As to the central nervous system diseases, intracerebral tumors play a primary role, and they could be represented by both head and neck cancers with intracranial spreading and trigeminal involvement (14–18).

The effects of the different therapies of cerebral tumors such as surgery (19–22) radiotherapy, (23) and systemic chemotherapy (24, 25) may also alter the nerve fibers or induce limbal stem cell deficiency (26, 27) resulting clinically in NK.

Association between diabetes and NK was identified already in 1977, as a consequence of the neuropathy of ophthalmic branch of trigeminal nerve due to microvascular damages of myelinated fibers (6, 28, 29).

The severity and progression of NK in diabetic patients are related to peripheral neuropathy, so the corneal nerve plexus is considered as an important marker of this latter's evolution and management effectiveness (6, 30). In particular NFL is considered a good parameter to evaluate the diabetic sensorimotor polyneuropathy (8).

Additionally, diabetes has further negative effects on the ocular surface inducing tear film instability and ocular surface microbiome alterations, that increase corneal erosion and infection susceptibility (31–33).

As to the other systemic diseases that may induce NK, the autoimmune diseases such as rheumatoid arthritis, Grave's disease are reported and were observed in 9% of patients in our group (34, 35).

Other systemic causes of NK are rare and are represented by amyloidosis, leprosis, CIPD, disseminated lymphangiomatosis, T-cell lymphoma, syringomyelia, vitamin B12 deficiency, HIV, and ischemic conditions like Wallenberg syndrome or cocaine snorting (36–50).

Furthermore, in pediatric patients with NK different genetic syndromes were described.

They comprehend above all HSN, congenital insensitivity to pain with anhidrosis, Gómez-López-Hernández syndrome, Goldenhar syndrome, congenital trigeminal nerve aplasia, and other more infrequent conditions like APS, familial dysautonomia, and Crisponi/CISS1 syndrome (34, 50–67).

In our study the severity of neurotrophic keratopathy interestingly appeared to be related to the patients' age. The patients with PED were younger with respect to the patients with ulcers. In a previous study, Roszkowska et al. (6, 8) demonstrated that the age is the most important element that influence corneal sub-basal nerve plexus (SBNP) nerves length, tortuosity and density. This could explain the finding that more severe corneal defects were registered in older patients who already have lower SBNP parameters. We therefore speculate that at the basis of the severity of NK there is a component of cellular tropism linked to the age and general condition of the patient.

The diagnosis of NK is based firstly on medical and surgical history of the patient to investigate all those conditions that may underlie the pathology (9, 68). Indeed, it is mandatory to consider both ocular and systemic treatments which the patient is undergoing, focusing on those that could damage corneal sensory innervation. Since NK is characterized by damage to the trigeminal sensory innervation, patients commonly do not experience any symptoms, making the diagnosis of NK particularly challenging (67). It is for the same reason that patients often see the specialist only in late phases of the disease when the pathology is already at an advanced stage (69, 70). It is important to perform a complete neurological assessment to reveal any cranial nerve damage, because a trigeminal nerve impairment may be associated with other cranial nerves injuries (1). NK's treatment consists of medical therapies, non-surgical and surgical intervention, depending on disease's stage (1, 10, 71–77).

Cenegermin is a recombinant human nerve growth factor, and it is the first EMA and FDA approved medication for moderate and severe NK in adults. It acts by promoting the growth of corneal nerves, differentiation, proliferation, and migration of corneal epithelial cells and maintaining corneal epithelial limbal stem cells (72–74).

Cenegermin resulted effective in different clinical studies on NK, but only few reports discussed its efficacy in disease related to systemic causes (72, 75–77). In this study, Cenegermin was effective in promoting corneal healing in both moderate and severe NK related to systemic diseases, with improvement of corneal sensitivity and complete recovery of the surface defects. Interestingly the severe forms healed faster with respect to PED. We reported the same results in our recent study when we analyzed the efficacy of Cenegermin in all patients with NK independently of underlying cause. About this we hypothesized that in ulcers the rh-NGF promotes better stromal healing with restore of the corneal thickness that induces the faster epithelial resurfacing as compared to PED (78–81).

This interesting finding needs further investigation to be confirmed on higher number of treated patients.

It can be concluded that this study emphasizes the crucial relation between NK and systemic diseases and particularly neurological diseases and diabetes emerged as main conditions. Given the high prevalence of these systemic diseases and their socio-economic impact, the prevention and a proper early management of NK is of high importance. We believe that accurate preventing, managing and monitoring of these systemic diseases, can help to reduce the risk of presenting of moderate and severe forms of NK.

Additionally, we demonstrated on our sample that despite the associated systemic pathology, the use of rh-NGF was equally effective in all studied subjects. However, further studies with a larger number of participants are necessary to understand better the relationships between NK presentation and systemic diseases.

## Data Availability Statement

The raw data supporting the conclusions of this article will be made available by the authors, without undue reservation.

## Ethics Statement

The studies involving human participants were reviewed and approved by Ethical Committee of the University Hospital of Messina. The patients/participants provided their written informed consent to participate in this study.

## Author Contributions

GO: conceptualization, writing, review, editing, and data analysis. AM: conceptualization, data collection, and supervisor. AV: data collection and analysis. LI: data collection and original draft preparation. CA, FF, and UC: conceptualization, writing, review, and editing. PA: conceptualization, writing, review and editing, original draft preparation, data analysis, and supervision. All authors contributed to the article and approved the submitted version.

## Conflict of Interest

The authors declare that the research was conducted in the absence of any commercial or financial relationships that could be construed as a potential conflict of interest.

## Publisher's Note

All claims expressed in this article are solely those of the authors and do not necessarily represent those of their affiliated organizations, or those of the publisher, the editors and the reviewers. Any product that may be evaluated in this article, or claim that may be made by its manufacturer, is not guaranteed or endorsed by the publisher.
